# The Presentation and Treatment of Myointimoma: A Systematic Review and the First Case Report of Penile Myointimoma as a Cause of Urethral Obstruction

**DOI:** 10.3390/jcm15031130

**Published:** 2026-02-01

**Authors:** Deirdre Maria König-Castillo, Armin Henning, Richard Wasicky, Clemens Kinsky, H. Christoph Klingler, Eva M. Compérat

**Affiliations:** 1 Department of Urology, Klinik Ottakring, 1160 Vienna, Austria; deirdre.koenig-castillo@gesundheitsverbund.at (D.M.K.-C.);; 2 Institute of Pathology, Klinik Ottakring, 1160 Vienna, Austria; 3 Department of Pathology, Medical University of Vienna, 1090 Vienna, Austria

**Keywords:** bladder outlet obstruction, myointimoma, penis, soft tissue tumor, urethral obstruction, urethral stricture

## Abstract

**Background/Objectives:** Myointimoma is a rare, benign soft tissue tumor of the penis. We present a systematic review of the current literature and a case report of a 33-year-old male with a urethral stricture without discernible risk factors. Our aim was to investigate current knowledge on Myointimomas and increase the awareness of this entity. **Methods**: A systematic literature search was conducted across EMBASE, MEDLINE, PubMed, Scopus, Web of Science, and Google Scholar. Only 30 previously reported cases of this tumor are known—all located at the glans penis or in close proximity to it. **Results**: After the exclusion of non-eligible studies, 14 studies were included. **Conclusions**: Myointimomas are a relevant differential diagnosis in recurring cases of bladder outlet obstruction.

## 1. Introduction

Benign tumors of the penis are a rare pathological entity. Thus, while the prevalence rates of malignant carcinomas of the penis in Europe are well documented (0.3–1.0 per 100.000 male population per year; responsible for 0.4–0.6% of all carcinomas in males), data on benign penile tumors are scarce, and no valid incidence rates are known [1]. Among these benign tumors, Myointimoma is an even more exceedingly rare, benign, multinodular proliferation derived from intimal cells of the corpus spongiosum’s vascular spaces [2].

To date, knowledge of the etiopathogenesis and possible risk factors remains limited, as data are still based on a limited number of cases. In previous cases, the patients were between 2 and 74 years old (median 32 years) with a tumor size from 0.4 to 1.9 cm (median 1 cm) [2,3,4,5,6,7,8,9,10,11,12,13,14,15]. The location was typically given as situated on the glans penis and corona of the glans. Commonly, an initial phase of rapid tumor growth was followed by a period of stable disease [2,3,4,6,7,8,9,10,11,13,14,15].

None of the patients reported any symptoms besides swelling.

Interestingly, no cases of recurrence after complete (or even incomplete) excision are known in the literature. One case of spontaneous remission occurred in a patient following a punch biopsy. Stable disease was seen in two patients after 6 and 10 months of follow-up [2]. Previous efforts to define risk factors for the development of Myointimoma have all failed to yield conclusive results (i.e., collagen vascular disease, diabetes mellitus, trauma, autoimmune disease, genitourinary infection, circumcision) [2,3,4,5,6,7,8,9,10,11,12,13,14,15].

## 2. Materials and Methods

### 2.1. Search Strategy and Data Sources

This systematic review followed the Preferred Reporting Item for Systematic Reviews and Meta-analyses (PRISMA) reporting guidelines [16]. A systematic literature search was conducted across EMBASE, MEDLINE, PubMed, Scopus, Web of Science, and Google Scholar. The literature search was performed without restrictions regarding publication year or language to ensure the comprehensive identification of all available evidence on this rare entity.

The following search strategy was applied (and adapted as necessary for each database):

(‘penile soft tissue tumor’ OR (penile AND soft AND (tissue OR tissue explosion) AND (tumor OR tumor explosion)) OR myointimoma) AND urology.

Both controlled vocabulary terms (e.g., Emtree terms in EMBASE) and free-text terms were used to maximize sensitivity. In addition, the reference lists of all included articles were manually screened to identify further relevant publications.

### 2.2. Eligibility Criteria

All studies reporting on Myointimoma of the penis or penile soft tissue tumors subsequently classified as Myointimoma were eligible for inclusion. Due to the rarity of the disease, case reports, case series, and clinicopathological studies were included. No restrictions were applied regarding the following: year of publication, language, or study design. Reviews were excluded from formal inclusion but were screened for relevant citations.

### 2.3. Study Selection

All identified records were imported into EndNote © (The EndNote Team 2013, EndNote 20 by Clarivate, Philadelphia, PA, USA), and duplicates were removed. Two reviewers independently screened all titles and abstracts, followed by a full-text review of potentially eligible studies.

Disagreements regarding study eligibility were resolved through discussion, with the involvement of a third reviewer in cases when consensus could not be reached. This procedure was applied consistently at both the abstract and full-text screening stages.

### 2.4. Data Extraction

Data extraction was performed independently by two reviewers using a predefined and standardized data extraction form. Extracted variables included the following:

Patient demographics, tumor localization, tumor size, clinical presentation, duration of symptoms, treatment modalities, clinical outcomes (including recurrence), follow-up duration, risk factors reported, country of origin of report, first author of report, and year of publication of report.

Any discrepancies in data extraction were resolved by consensus or, if necessary, by consultation with a third reviewer.

### 2.5. Data Synthesis

Due to the limited number of cases available, a qualitative narrative synthesis was conducted, but no meta-analysis was performed.

## 3. Results

### 3.1. Study Selection

The systematic literature search identified a total of 648 records across six databases. After removing 221 duplicate records, 427 records were screened based on their titles. Following title screening, 397 records were excluded. The abstract screening of the remaining 30 records led to the exclusion of 7 additional studies.

**Figure 1:**
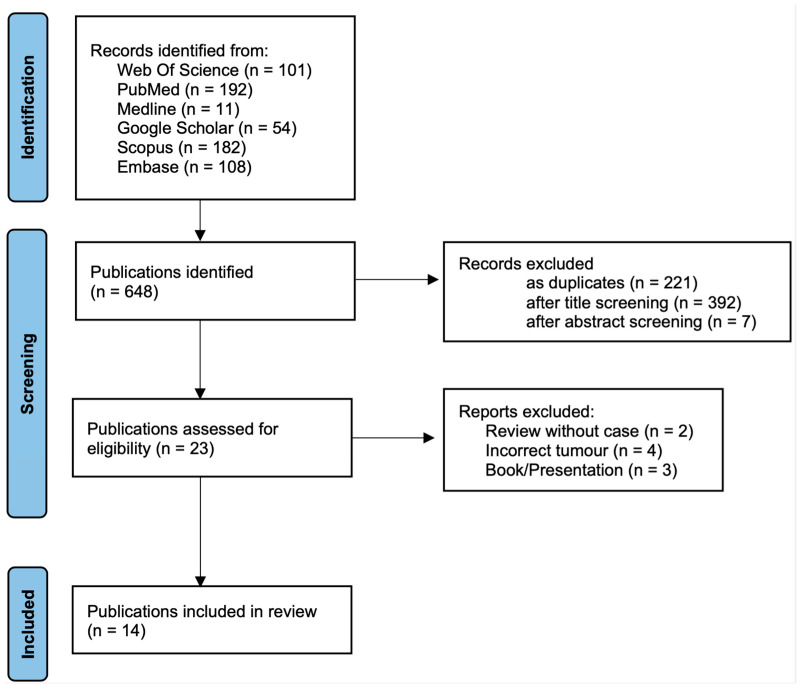
A flow chart of the articles included in this systematic review according to the PRISMA guidelines by Page, MJ et al. (2020) [16].

A total of 23 full-text articles were assessed for eligibility. Of these, nine articles were excluded due to the absence of original cases (n = 2), incorrect tumor entity (n = 4), or failure to meet inclusion criteria (n = 3). A total of 14 studies were included in the qualitative synthesis. The study selection process is summarized in a PRISMA flow diagram (Figure 1). For the PRISMA checklist, see the Supplementary Materials.

### 3.2. Study Characteristics

All included publications were case reports (n = 11) or small case series (n = 3 with 10, 5, and 4 cases, respectively) describing 30 patients with histologically confirmed Myointimoma of the penis. The identified cases were published between 2000 and 2023 and originated from diverse geographic regions (Table 1).

### 3.3. Clinical Presentation and Diagnostic Workup

A total of 29 of the 30 reported Myointimoma cases were located at the glans penis or corona of the glans penis. Clinical presentation was variable but typically involved an initial rapid growth followed by stable disease and a painless penile mass. Diagnostic evaluation commonly included physical examination [2,3,4,6,7,8,9,10,11,12,13,14,15]. Only one case presented with a Myointimoma located at the ventral portion of the penis, next to the glans. Additionally, this case showed a divergent growth characteristic: after a stable phase of 30 years, a rapid expansion in size was observed [5].

All patients underwent local surgical excision as primary treatment. Of the 30 cases, only 13 were treated with a complete excision of the tumor. In 12 of the cases, the tumors were treated with an incomplete excision. In four cases, information was not fully available [2,3,4,5,6,7,8,9,10,11,12,13,14,15].

Definitive diagnosis in all included cases was established by histopathological examination, frequently supported by immunohistochemical analysis [2,3,4,5,6,7,8,9,10,11,12,13,14,15].

Interestingly, Wyner et al. reported the case of a 69-year-old male patient with an uncommon histology. He had previously been diagnosed with prostate cancer and treated by external beam radiotherapy. Due to his diabetic bladder dysfunction, he was performing intermittent self-catheterization. Except for a hemorrhagic cystitis requiring multiple blood transfusions, he showed stable disease regarding prostate cancer. After several years of nearly stable prostate-specific antigen (PSA) levels, a gradual rise to 1.11 ng/mL was noted (post-treatment nadir 0.27 ng/mL). At a regular checkup, he presented with a newly occurring fleshy mass on the glans. The mass was locally excised, and a circumcision was performed sparing the penis. The mass showed a bigger part of 1.0 cm and a smaller base part with 0.4 cm. Interestingly, the histology of the bigger part showed a mix of fibroepithelial polyp and a Myointimoma. Meanwhile, the histology of the base showed a Gleason 4 + 4 metastatic ductal prostatic adenocarcinoma [15].

Another interesting case with a divergent tumor location and growth pattern was described by Cordeiro et al., who presented the case of a 50-year-old patient with a small nodule on the ventral portion of the penis next to the glans. The tumor size had been stable for 30 years, followed by rapid growth. Thereafter, a total excision was performed (tumor size 1.5 cm). During the complete follow-up of 6 months, there was no evidence of disease [5].

### 3.4. Histopathological and Immunohistochemical Findings

Histologically, Myointimoma is characterized by multinodular, intravascular proliferation within the vascular intima of the corpus spongiosum. At higher magnification, spindle-shaped and stellate cells are observable.

Immunohistochemically, the cells were positive for smooth muscle actin and calponin but negative for desmin, cytokeratins, CD34, and the S-100 protein [2,3,4,5,6,7,8,9,10,11,12,13,14,15].

### 3.5. Treatment and Outcomes

Across the included studies, no aggressive behavior or metastatic disease was reported. While follow-up durations varied (from 1 month to 15 years), no recurrences were documented in cases with available follow-up data. Of the reported 30 cases, 24 showed no evidence of disease at the end of follow-up, while 2 showed stable disease after incomplete excision [2,3,4,5,6,7,8,9,10,11,12,13,14,15]. Interestingly, even one case of a 10-year-old boy showed a spontaneous remission after the punch biopsy of a 1.0 cm tumor. However, in three cases, information was not fully available [2].

## 4. Case Report

In addition to the conducted systematic review, we report on the case of a urethral Myointimoma as a rare cause of obstructive voiding symptoms due to urethral stricture in an otherwise healthy 33-year-old European male of African descent.

The patient had been referred to our department, a tertiary referral center, after prior evaluation at the primary care level due to complaints of lower urinary tract obstruction and an impaired urinary flow, and elevated residual urine had been found.

He reported a history of voiding symptoms, including slow stream, hesitancy, dysuria, and straining, reaching back several months. No risk factors for urethral stricture formation (i.e., prior transurethral surgery, urethral manipulation, trauma, or urethritis) were present in the patient’s medical history.

**Figure 2:**
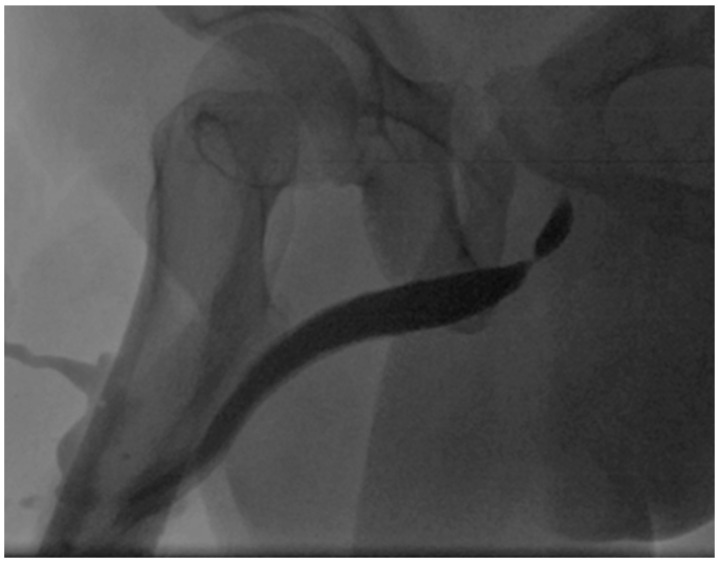
Bulbar urethral stricture of 2 cm in length.

At our department, a urinary flow measurement (uroflow) and a urethrocystography (UCG) were performed. The uroflow showed an obstructive, protracted, plateau-shaped voiding curve with a maximal urinary flow (q/max) of 5ml/sec at 255mL of voided volume. UCG revealed a short, bulbar urethral stricture of 2cm in length (see Figure 2).

Subsequently, the patient was scheduled to undergo direct visual internal urethrotomy (DVUI), according to Sachse, which was—due to temporary limitations in anesthesia capacities during the SARS-CoV-2 pandemic—completed at another institution. During the cystoscopy, the tissue appeared macroscopically unsuspicious, and nothing uncommon had been apparent. The DVUI was performed without complication, and the patient was discharged after two days of inpatient hospital stay and a successful trial without catheter (TWOC).

Over the following years, the voiding symptoms had subsided, and regular primary care checkups revealed neither residual urine nor impaired urinary flow.

**Figure 3:**
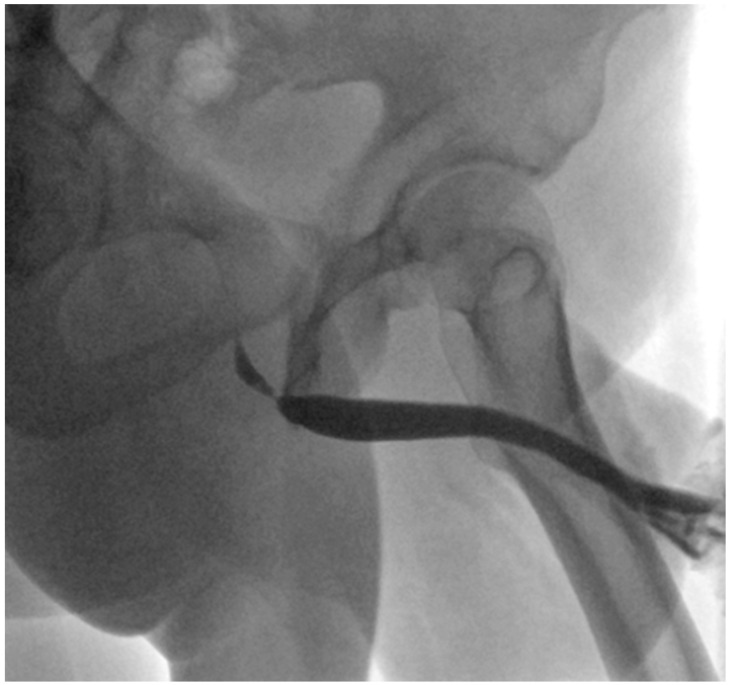
Recurring short bulbar stricture at the exact same location.

However, three years after the initial presentation, the patient returned to our department due to a relapse of voiding difficulties. Urethrocystoscopy and UCG (see Figure 3) confirmed the recurrence of a short bulbar stricture at the same location as three years prior. At this point, several options were discussed, including another attempt at endoluminal treatment. However, following current EAU guidelines for short recurrent bulbar urethral strictures, the patient opted for a non-transecting excision and primary anastomosis (ntEPA).

Urethroplasty was performed in the autumn of 2024. Surgery was complicated due to an unusually high amount of urethral bleeding, which had been difficult to manage intraoperatively. Hence, the non-transecting approach had to be abandoned, and a complete thickness resection of the bulbar urethral segment holding 2 cm in length and reconstruction by a tension-free spatulated anastomosis using eight monofilament interrupted sutures (Ethicon Monocryl 4-0^®^) was performed. The operating time was 119 min, and intraoperative blood loss was 300 mL. However, postoperative recovery was uneventful, and the patient was discharged from the hospital on postoperative day 11 after a TWOC showing unimpaired voiding, unimpaired urinary flow, no residual urine, and a regular UCG.

**Figure 4:**
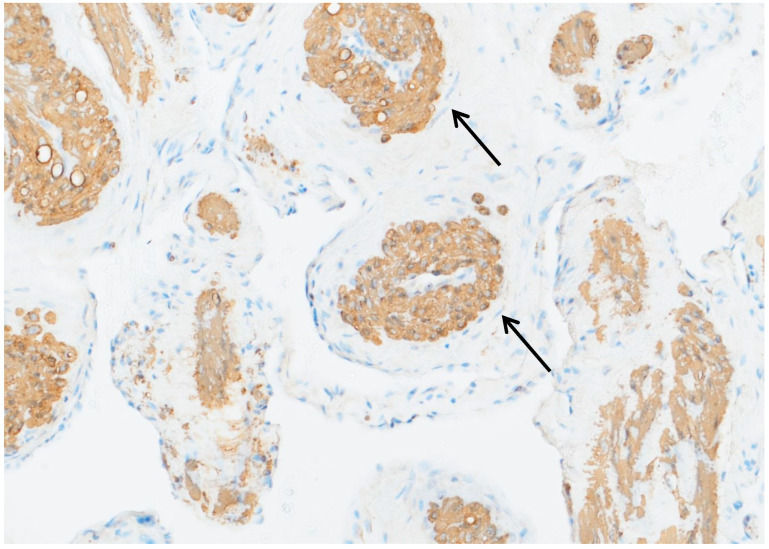
Immunohistochemistry: anti-smooth muscle actin. A magnification of ×20 was used. This figure shows the typical multilocular, intravascular myointimal proliferation with the vascular lumen in the center (examples indicated by the black arrows).

**Figure 5:**
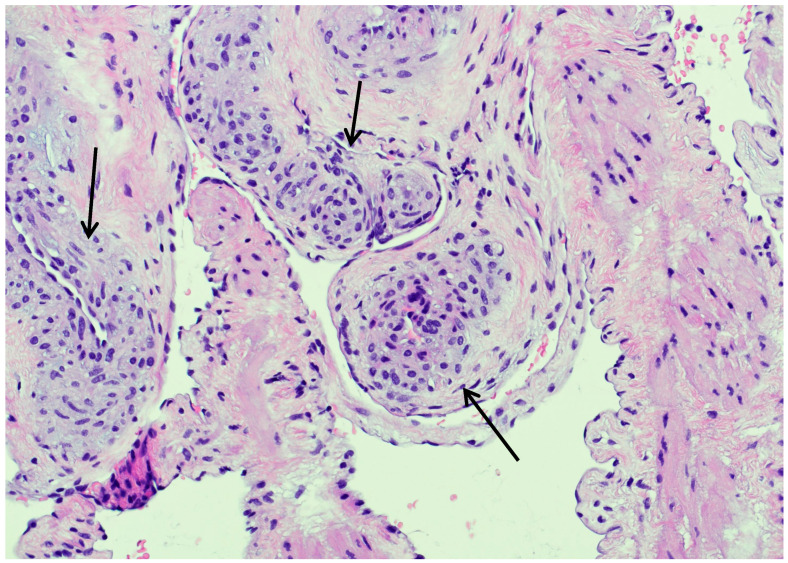
Hematoxylin and eosin staining. A magnification of ×20 was used. This figure shows the typical nodular architecture, for Myointimoma, with predominating spindle cells (examples indicated by the black arrows).

Following histopathological analyses, a Myointimoma—to the best of our knowledge, the first case of a Myointimoma in the urethra ever described in the literature—was diagnosed. Histology revealed diffuse, multifocally distributed clusters of spindled cell elements just beyond the endothelial layer of the vascular structures of the penile corpus spongiosum. The nuclei were described as round to elongated and cytologically bland, with only minimal mitotic activity (see Figure 4 and Figure 5). Immunohistochemically, cells typically expressed SMA, but stainings for S100 and CD 34 were negative.

**Figure 6:**
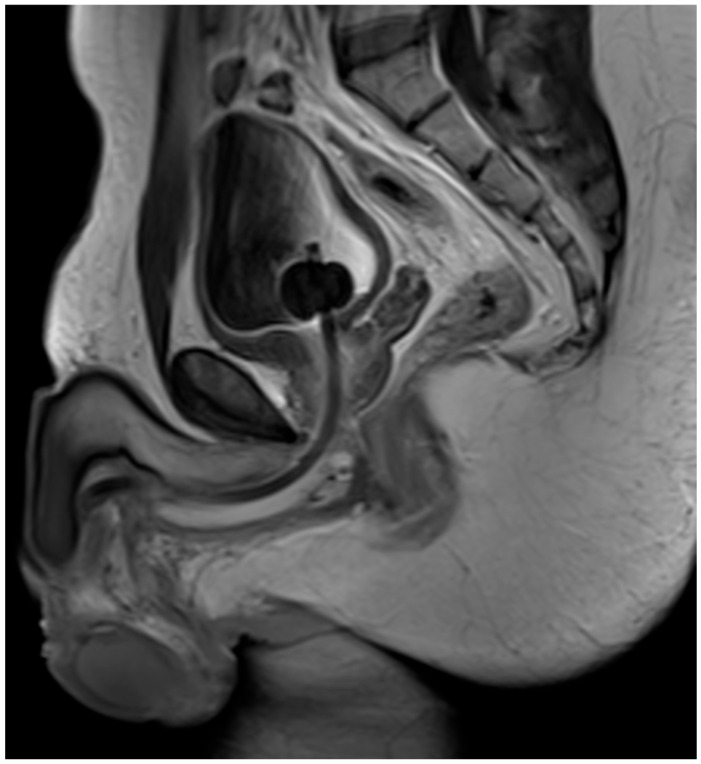
Follow-up MRI found no evidence of tumor recurrence, dehiscence, or fistula.

On postoperative day 27, the patient reported perineal urine loss during voiding on clinical checkup, caused by an anastomotic dehiscence and perineal urinary fistula formation. Under cystoscopic and guide-wire guidance, a 16 French Foley Catheter was placed. On postoperative day 52, a contrast series MRI confirmed no evidence of tumor recurrence, dehiscence, or fistula (see Figure 6).

The indwelling catheter was removed on postoperative day 104. On further follow-up, voiding was asymptomatic, urinary flow was unimpaired, and the UCG was regular, showing no signs of stricture recurrence or fistula. Furthermore, the patient never reported any erectile dysfunction before or after the operation. As of March 2025, the patient is in clinical follow-up with no signs of recurrence.

## 5. Discussion

Myointimoma is a rare, benign tumor of the penis first described by Fetsch et al. in 2000 in a retrospective case series [2].

The clinical behavior of Myointimoma is of high interest—especially given the tumor’s unorthodox behavior. Typically, after an initial phase of rapid progression, growth seems to be suspended thereafter [14]. However, one case of late growth progress after 30 years of stable disease was reported [2]. Long-term stable disease and even spontaneous remission have been described, whereas aggressive growth and metastases are unknown [2].

Nevertheless, due to the small number of published cases, definitive knowledge of risk factors, growth patterns, location, or therapeutic options remains scarce. As Myointimoma is a relatively unknown entity with few or no symptoms, it may be underdiagnosed.

Considering that 15% of penile strictures and even 40% of bulbar strictures, thus far, are idiopathic (e.g., the cause of the stricture is not known) [17], and histological workup does not always occur in these cases, increased awareness of Myointimoma as a relevant differential diagnosis may reveal more cases.

Despite ongoing efforts, no risk factors for the development of a Myointimoma could be identified. A genetic analysis could give further insights.

To the best of the authors’ knowledge, we report the first case of a Myointimoma causing a bladder outlet obstruction and lower urinary tract symptoms.

Unusual constellations, such as an unclear medical history, unusual (imaging) findings, or high intraoperative blood loss during stricture surgery, should be understood as an indicator for, possibly, unusual causes of urethral stricture formation which may affect clinical outcome (as shown in the presented case with the Myointimoma causing a high amount of blood loss and the formation of a fistula). In such unusual cases, a histological workup should be considered. In patients with unclear constellations, considering rare causes and taking a histology might be helpful in clinical diagnosis, possibly affecting surgical procedures and allowing valuable information to be obtained about urethra strictures or tumors in the future.

The following limitations should be considered in the context of these interpretations: As Myointimoma is still a relatively unknown entity, only a very limited number of previous publications were available. Thus, no meta-analysis could be performed. Furthermore, sensitivity analyses or analyses on the risk of bias could not be performed. However, this only underscores the necessity of an increase of awareness in the possible differential diagnosis of Myointimoma in unclear cases.

## 6. Conclusions

Although a rare entity, increased awareness of Myointimomas and other benign tumors of the penis as potential differential diagnoses may affect procedures and improve patient outcomes.

To improve clinical care and surgical outcome, it is important to consider soft tissue tumors in urethral strictures in patients without any risk factors for urethral strictures. Additionally, it is important to find risk factors for Myointimoma or other benign soft tissue tumors of the penis. Furthermore, considering differential diagnoses is important to identify more aggressive tumors and prevent a severe course of disease.

## Figures and Tables

**Table 1:** Reported cases of Myointimoma, including their clinicopathologic features, the authors of the case report, the year of publication, and the age of the patient at the time of diagnosis given in years.

Age (Years)	Location	Size (cm)	Durtion	Therapy	Follow-Up	Out-Come	Country	Author	Publication (Year)	DM	Trauma	Collagen-Vascular Disease	Autoimmune Disease	Genitourinary Infection
2	Glans	0.5	NA	complete resection	NA	NA	USA	Fetsch	2000	non	non	non	non	non
2	Glans	1.0	NA	punch biopsy	10 yrs	Remission	USA	Fetsch	2000	non	non	non	non	non
4	Corona of glans	0.7	4-5 mos	complete resection	1 yr 7 mos	NED	USA	Fetsch	2000	non	non	non	non	non
19	Glans	1.1	>6 mos	complete resection	NA	NA	USA	Fetsch	2000	non	non	non	non	non
30	Glans	0.6	3 wks	complete resection	6 yrs 5 mos	NED	USA	Fetsch	2000	non	non	non	non	non
32	Glans	0.7	4 wks	Incomplete excision	6 mos	Persistent/stable disease	USA	Fetsch	2000	non	non	non	non	non
37	Corona of glans	1.1	NA	complete resection	13 yrs	NED	USA	Fetsch	2000	non	non	non	non	non
53	Corona of glans	1.9	4 wks	complete resection	5 yrs	NED	USA	Fetsch	2000	non	non	non	non	non
54	Glans	0.9	4 days	complete resection	9 yrs 9 mos	NED	USA	Fetsch	2000	non	non	non	non	non
61	Corona of Glans	0.8	5 mos	complete resection	3 mos	NED, tenderness	USA	Fetsch	2000	non	non	non	non	non
54	Corona of glans	0.4	2 mos	Incisional biopsy	1 mo	NED	USA	Robbins	2005	non	non	non	non	non
12	Glans	0.4	NA	marginal excision	7 mos	NED	USA	McKenney	2007	non	non	non	non	non
4	Glans	0.7	NA	marginal excision	3 yrs 9 mos	NED	USA	McKenney	2007	non	non	non	non	non
9	Glans	0.5	NA	incomplete excision	1 yr 9 mos	NED	USA	McKenney	2007	non	non	non	non	non
15	Glans	1.8	NA	incomplete excision	2 mos	NED	USA	McKenney	2007	non	non	non	non	non
9	Glans	1.0	NA	incomplete excision	1 yr 6 mos	NED	USA	McKenney	2007	non	non	non	non	non
50	ventral portion of the penis; next to the glans	1.5	30 yrs	complete excision	6 mos	NED	Brazil	Cordeiro	2007	non	non	non	non	non
50	Glans	1.0	2 mos	excisional biopsy	9 mos	NED	Turkey	Vardar	2007	non	non	non	non	non
54	Corona of glans	0.4	3 mos	incisional biopsy	1 mo	NED	USA	Thurber	2008	NA	non	NA	NA	NA
14	Glans	1.0	1 mo	excisional biopsy	NA	NA	USA	Turner	2009	non	non	non	non	non
74	Glans	1.0	4 mos	NA	10 mos	persistent/stable disease	Spain	Mossálvez	2009	non	non	non	non	non
53	Glans	0.8	NA	complete excision	6 mos	NED	Czech Repuplic	Tolinger	2014	non	non	non	non	non
69	Glans	1.0	22 mos	excision	3 mos	NED	USA	Wyner	2016	yes	yes	non	non	NA
11	Glans	1.0	2 wks	complete excision	1 yr	NED	Turkey	Tanriverdi	2019	NA	non	NA	NA	non
49	Glans	1.1	1 yr	complete excision	1 yr 6 mos	NED	Italy	Cito	2020	non	non	non	non	non
15	Glans	1.0	6 mos	excisional biopsy	3 yrs	NED	Czech Republic	Drlík	2022	non	non	non	non	non
20	Glans	0.9	2 wks	NA	9 mos	NED	USA	Casa	2023	non	non	non	non	non
42	Glans	NA	NA	NA	11 yrs	NED	USA	Casa	2023	non	non	non	non	non
32	Glasn	NA	NA	NA	4 yrs 8 mos	NED	USA	Casa	2023	non	non	non	non	non
68	Glans	NA	NA	NA	15 yrs	NED	USA	Casa	2023	non	non	non	non	non

Abbreviations: NA: not available; NED: no evidence of disease; yr(s): year(s); mo(s): month(s); wks: weeks; DM: diabetes mellitus.

## Data Availability

Data is contained within the article or Supplementary Materials.

## Supplementary Materials

The following supporting information can be downloaded at: https://www.mdpi.com/article/10.3390/jcm15031130/s1, PRISMA 2020 Main Checklist.

## Abbreviations

The following abbreviations are used in this manuscript:

PSA	prostate-specific antigen
UCG	urethrocystography
DVUI	direct visual internal urethrotomy
TWOC	trial without a catheter
ntEPA	non-transecting excision and primary anastomosis
uroflow	urinary flow measurement
q/max	maximal urinary flow
NA	not available
NED	no evidence of disease
Yr(s)	year(s)
MO	months
Wks	weeks
D	day(s)
DM	diabetes mellitus
